# Lipid species affect morphology of endoplasmic reticulum: a sea urchin oocyte model of reversible manipulation[S]

**DOI:** 10.1194/jlr.RA119000210

**Published:** 2019-09-23

**Authors:** Gabriela Ulloa, Fadi Hamati, Alexander Dick, Julie Fitzgerald, Judith Mantell, Paul Verkade, Lucy Collinson, Kenton Arkill, Banafshe Larijani, Dominic Poccia

**Affiliations:** Department of Biology,* Amherst College, Amherst, MA; School of Biochemistry,† University of Bristol, Bristol, United Kingdom; Francis Crick Institute,§ London, United Kingdom; School of Medicine,** Faculty of Medicine and Health Sciences, University of Nottingham Medical School, Nottingham, United Kingdom; Centre for Therapeutic Innovation,†† Cell Biophysics Laboratory, Department of Pharmacy and Pharmacology and Department of Physics, University of Bath, Claverton Down, Bath, United Kingdom, and Cell Biophysics Laboratory, Ikerbasque, Basque Foundation for Science, Research Centre for Experimental Marine Biology and Biotechnology (PiE) and Biophysics Institute (UPV/EHU, CSIC), University of the Basque Country, Leioa, Spain

**Keywords:** membrane lipids, diacylglycerol, lipid kinases, phospholipases, negative spontaneous curvature, serial blockface scanning electron microscopy, electron tomography, confocal microscopy

## Abstract

The ER is a large multifunctional organelle of eukaryotic cells. Malfunction of the ER in various disease states, such as atherosclerosis, diabetes, cancer, Alzheimer’s and Parkinson’s and amyotrophic lateral sclerosis, often correlates with alterations in its morphology. The ER exhibits regionally variable membrane morphology that includes, at the extremes, large relatively flat surfaces and interconnected tubular structures highly curved in cross-section. ER morphology is controlled by shaping proteins that associate with membrane lipids. To investigate the role of these lipids, we developed a sea urchin oocyte model, a relatively quiescent cell in which the ER consists mostly of tubules. We altered levels of endogenous diacylglycerol (DAG), phosphatidylethanolamine (PtdEth), and phosphatidylcholine by microinjection of enzymes or lipid delivery by liposomes and evaluated shape changes with 2D and 3D confocal imaging and 3D electron microscopy. Decreases and increases in the levels of lipids such as DAG or PtdEth characterized by negative spontaneous curvature correlated with conversion to sheet structures or tubules, respectively. The effects of endogenous alterations of DAG were reversible upon exogenous delivery of lipids of negative spontaneous curvature. These data suggest that proteins require threshold amounts of such lipids and that localized deficiencies of the lipids could contribute to alterations of ER morphology. The oocyte modeling system should be beneficial to studies directed at understanding requirements of lipid species in interactions leading to alterations of organelle shaping.

The ER is an organelle composed of a lumen enclosed by a single membrane that extends throughout the cytoplasm of eukaryotic cells. Among the functions associated with this organelle are lipid synthesis, Ca^2+^ signaling, detoxification reactions, and processing, folding, synthesis, transport, and membrane insertion of proteins (1, 2). Disruption of protein folding mechanisms in mutant cells leads to ER stress triggering an unfolded protein response, which may result in protein aggregation or apoptosis (3, 4). Prolonged stress has been associated with a number of human diseases, such as viral hepatitis, hereditary spastic paraplegias, atherosclerosis, type 2 diabetes, and cancer (5, 6), and various neurological and neurodegenerative diseases, such as Alzheimer’s, Parkinson’s, and amyotrophic lateral sclerosis. These diseases are often accompanied by changes in ER morphology. Morphological changes in the ER have also been linked in *Drosophila* to neural synapse malfunction (7).

The ER is a dynamic and continuous anastomosing structure consisting of two extreme and often dynamic morphs, tubules and sheets, of overall high and low degrees of curvature, respectively (1, 8), and variable amounts of intermediate structures such as fenestrated sheets and fenestrated networks, which are difficult to assign to either sheets or AL and may represent transitional structures in sheet/tubule interconversions (9). The network can be remodeled by fusion and budding, for example during cell cycle progression from interphase to mitosis (10) or under various conditions during interphase (11). Specialized cells illustrate a general functional distinction between sheets and tubules. Generally, cells synthesizing large amounts of secreted or integral membrane proteins, such as β-pancreatic cells, are replete with sheets, often stacked (12). Cells synthesizing large amounts of lipids or exhibiting rapid and widespread Ca^2+^ signaling or extensive detoxification, such as steroid secretory cells, muscle, or liver, typically have a paucity of sheets but numerous tubules. The ER also forms specialized junctions of contact with mitochondria, plasma membranes, or lysosomes to facilitate lipid transfer (13).

Abundant evidence suggests that ER morphology is dependent on proteins that help to shape the membranes (1, 13–15). These include: *1*) tubule-forming proteins such as reticulons and DP1/Yop1/REEP5; *2*) GTPases like atlastin and Sey1p/RHD3 that facilitate membrane fusion; *3*) proteins that space the lumens of sheets (30–50 nm) such as Climp-63, kinectin, and p180; and *4*) various regulators like Lunapark proteins, protrudin, and Rab 10 and 18. Microtubule/ER interactions are also important for ER tubule formation and maintenance (16).

The contribution of membrane lipids to organelle shape maintenance or fusion has received much less attention than membrane proteins. We and others have emphasized the importance of the phospholipid composition of ER membranes in both ER shaping and membrane fusion (17–23). Even protein-free lipid bilayers can exhibit fusion facilitated by certain lipids, suggesting that the role of lipids in the ER is not entirely passive (24, 25).

Here, we report the dependence of ER structure on membrane lipid content in live mature oocytes of the sea urchin. These large cells are relatively metabolically inactive until fertilization. In the unfertilized egg, few polyribosomes are associated with rough ER, a characteristic of protein synthesis (26); although the egg’s low levels of protein synthesis are rapidly increased following fertilization with a 30-fold recruitment of ribosomes into polyribosomes (27). ER structure in the eggs is morphologically stable for long periods prior to fertilization.

The oocytes are highly transparent making them ideal for confocal microscopy. Their ER can be specifically labeled for confocal microscopy by microinjection of an oil droplet containing 1,1′-dioctadecyl-3,3,3′,3-tetramethylindocarbocyanine perchlorate (diIC_18_), a stable fluorescent membrane marker (11). Previous transmission electron microscopy (TEM) has revealed a paucity of sheets and stacks in these oocytes with the exception of the poorly understood annulate lamellae, sheet-like structures that resemble fragments of nuclear envelope easily distinguished by their regularly spaced structures resembling nuclear pores. Soon after fertilization, the annulate lamellae break down (26) and most of the tubular network is reorganized between 2 and 8 min (11).

To test the dependence of ER membrane morphology on lipids, we depleted or augmented specific phospholipid contents in unfertilized eggs. We previously showed that enzymatic depletion of 1,2-diacylglycerol (DAG) in these eggs resulted in creation of areas that appeared to contain sheet-like structures (20). These areas could be prevented from forming by exogenous delivery of lipids like phosphatidylethanolamine (PtdEth) or 1,3-DAG, a nonsignaling isomer of 1,2-DAG. Here, we show that, once started, development of these regions could be reversed by these phospholipids. The regions depended on a balance of endogenous 1,2-DAG depletion and formation, which we experimentally manipulated by varying the ratio of co-injected DAG kinase (DGK) and PtdIns-specific phospholipase C (PI-PLC). We also characterize here the detailed structure of the sheet regions by confocal microscopy and serial block-face scanning electron microscopy (SBF-SEM) (28). These regions consisted of an accumulation of extensive individual and stacked sheets and exclusion of yolk granules. We discuss possible mechanisms by which ER tubules may be reversibly converted to sheets by alteration of cellular phospholipid content.

## MATERIALS AND METHODS

### Chemicals

The chemicals used were Millipore-filtered sea water, DGK (Sigma-Aldrich D3065), PI-PLC (Sigma-Aldrich P5542), LB buffer as previously described (20), L-α-phosphatidylcholine (Egg PC) (Avanti 840051), L-α-phosphatidylethanolamine (Egg PE) (Avanti 110581), 1,3(d5)-di-(9Z-octadecenoyl)-glycerol (DAG) (Avanti 840021). LB loading buffer was 10 mM HEPES (pH 8.0), 250 mM NaCl, 25 mM EGTA, 5 mM MgCl_2_, 110 mM glycine, 250 mM glycerol, and 1 mM dithiothreitol. Millipore-filtered sea water was artificial sea water (Instant Ocean) filtered through a 0.22 μm Millipore GS filter.

### Animals

Adult *Lytechinus pictus* sea urchins were purchased from South Coast Bio-Marine, San-Pedro, CA, maintained in 15°C artificial sea water tanks with adequate aeration, and fed kelp each week. Eggs were collected by 10 V electrical stimulation across the body and the animals were returned to the tanks.

### Labeling of ER

Handling of oocytes and microinjection of diIC_18_ (Sigma-Aldrich 468495) has been previously described (20). Briefly, dejellied eggs were attached to protamine sulfate-coated coverslips attached to seal a hole in the bottom of a Petri dish (29). Microinjections were accomplished with an Eppendorf Transjector 5246 and Micromanipulator 5171. Aqueous injection of enzymes (DGK, PI-PLC) was fluorescently monitored by inclusion of 10 mg/ml FITC Dextran (Sigma-Aldrich 32H0451) in the loading buffer.

### Treatment with SUVs

Small unilamellar vesicles (SUVs) were prepared by sonication from aqueous suspensions of phospholipids as previously described (20). Preincubation of eggs with SUVs was for 30 min in sea water and washed out with sea water prior to microinjections. For reversal experiments, SUVs were added directly to eggs under the microscope to the same final concentrations as for preincubation.

### Injection of enzymes

DGK was injected after dilution with LB of a stock solution of 1 mg/ml in LB to final concentrations of 250, 100, or 50 μg/ml. PI-PLC was injected after dilution of a stock solution of 1 mg/ml (3.3 U/ml) to 200, 100, 10, or 5 μg/ml. Eggs damaged by injection were excluded from analyses.

### Inhibitors

ER-labeled unfertilized eggs were pulsed with the microtubule inhibitor, colcemid (Sigma 10295892001), final concentration 5 μM in Millipore-filtered sea water from a 2 mM stock, applied 8 min prior to DGK injection. A 10 min treatment with 5 μM of colcemid blocks microtubules for 2 h in sea urchin eggs, which is sufficient to block microtubule assembly for 2 h (30, 31) or continuously for up to 2 h.

To alter microfilament organization, the microfilament inhibitor, cytochalasin D (Sigma C8273), in sea water was added for 45 min (final concentration 4 μg/ml from 1 mg/ml stocks in DMSO) or eggs were pulsed for 15 min, conditions reported to impair cytokinesis in fertilized eggs and disrupt microfilaments (32, 33).

To block protein synthesis, emetine (Calbiochem, 32469) was added to eggs in sea water [final concentration 100 μM, sufficient to block by 95% in eggs up to 1 h (34)] for 30 min prior to injecting with DGK.

### Confocal microscopy

**Fig. 1.**
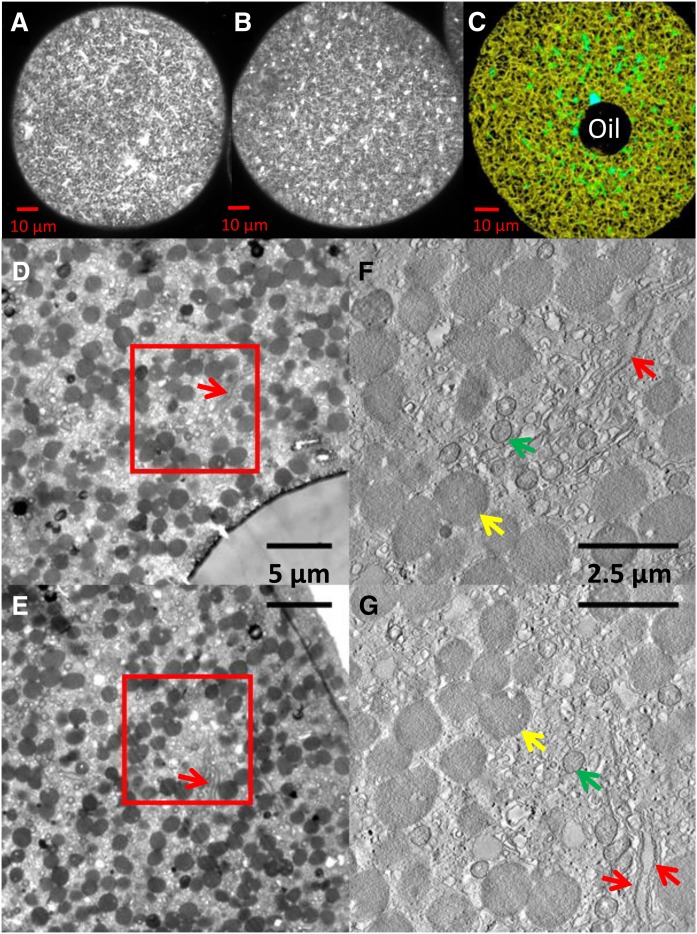
ER of unfertilized egg. A: Confocal microscopy image 30 min after microinjection of oil containing diIC_18_. B: Five hours after diIC_18_ injection illustrating similar pattern of ER. C: 3D reconstruction of egg made from z-stack of six 0.1 μm optical sections analyzed with Microscopy Image Browser. Total ER was selected with a Strel filter and labeled yellow, brighter sheet-like areas (selected by black/white intensity thresholding) were labeled turquoise and superimposed. Tubular density is apparent. D, E: Representative transmission electron micrographs of 300 nm sections showing the injected oil (D) and egg edge (E). F, G: Tomographic slices from the boxed areas in D and E. Yolk granules (yellow arrows) appear relatively uniformly distributed throughout the cytoplasm. In areas with fewer yolk granules, annulate lamellae (red arrows), mitochondria (green arrows), membrane vesicles, ER tubules, and rare ER sheets can be seen.

**Fig. 2.**
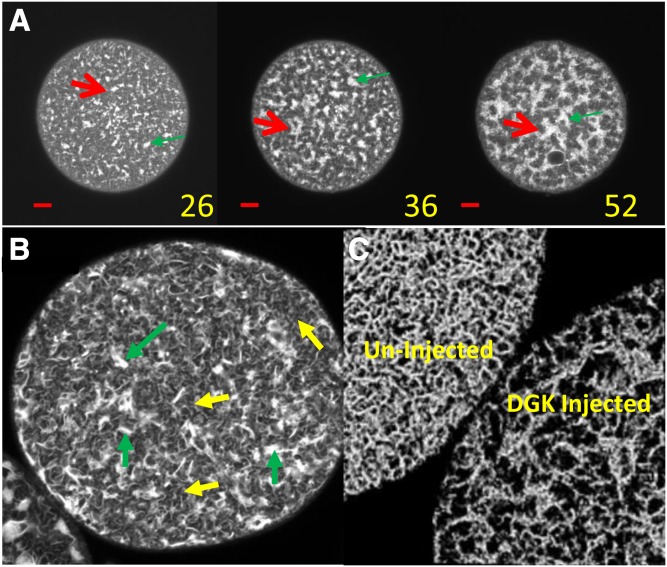
ER of DGK-treated eggs. A: Confocal images of an egg injected with DGK showing accumulation of sheet regions at 28, 36, and 52 min post-DGK injection. Green arrows indicate representative sheet regions. Red scale lines = 10 μm. B: Confocal image of an unlabeled egg injected with DGK and 20 min later injected with diIC_18_ to test for ER continuity. Green arrows indicate representative sheet areas; yellow arrows indicate representative tubular areas. Sheets and tubules remain connected. C: Confocal 3D reconstruction of control and DGK-treated eggs made from z-stack of 18 overlapping 0.1 μm sections (total depth 0.9 μm) and analyzed with Microscopy Image Browser using a Strel filter to select for the tubular network (Strel 4, b/w threshold 0.04, size limit 70) and Fiji volumes showing depletion of tubules by DGK.

**Fig. 3.**
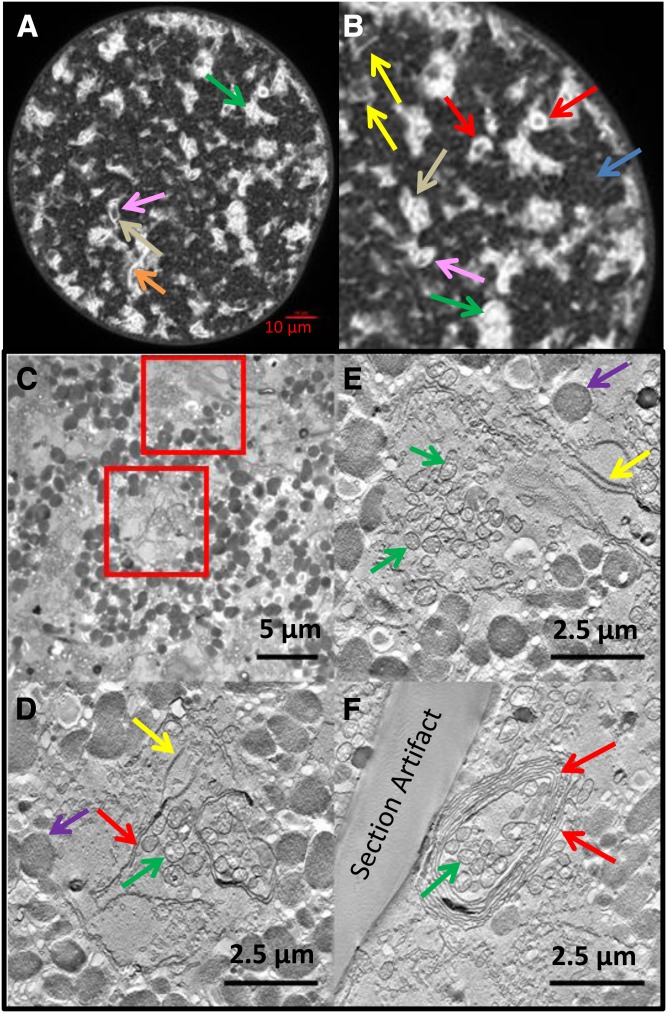
The ER displays a variety of structures in sheet regions. A: Confocal image 80 min after DGK injection shows bright sheet-like areas against background of more faint connecting tubules with extensive sheet formation illustrating many structures confirmed by electron microscopy. B: Enlargement of A. Arrows indicate edge views of sheets or thick stacks (yellow), thicker stacks (orange), partially closed stacks (pink), closed stacks/cylinders (red), bifurcations of stacks (tan), en face views of sheets possibly fenestrated (green), area of tubules (blue). C: EM section of DGK-treated egg. D, E: Transmission electron micrograph of 300 nm sections with planes from tomographic reconstructions of the two boxed areas shown in C. Note sheets and fenestrated sheets extending from annulate lamellae. E: Note prominent annulate lamellae and fenestrated sheet areas. F: A coiled stacked sheet from elsewhere in the same egg. D–F: Yolk granules (purple arrows), mitochondria (green arrows), fenestrated sheet/annulate lamellae (yellow arrows), nonfenestrated sheets (red arrows).

Images were obtained with a Nikon Eclipse-Ti confocal microscope and NIS Elements software using either 40× (NA 1.3), 60× (NA 1.4), or 100× (NA 1.4) objectives. Time sequences or z-stacks were acquired. The distinction between tubules and sheet regions was based on relative fluorescence intensity from time series images taken at 40×, which improves the contrast between these regions (see Figs. 1A, B; 2A; 3A, B). The analysis in Fig. 1C is the basis for quantifications shown in the graphs where the total ER fluorescence above background is taken as the total ER in the single plane of a single time point and the intensity cutoff is determined manually for larger objects representing sheet areas. Post-acquisition analysis was with Volocity software (v 6.3; Perkin-Elmer) or Microscopy Image Browser (http://mib.helsinki.fi/) with MatLab R2016a (MathWorks) and FIJI [ImageJ (35)] installed.

### Sample preparation for electron microscopy

ER-labeled eggs (stuck to either regular coverslips or gridded coverslips from Bellco Glass, Inc., Vineland, NJ) cleaned in Alconox detergent, thoroughly washed in distilled water, ethanol washed, air dried, and microinjected with DGK were monitored for an appropriate time prior to a prefixation for 30 min in 0.1% paraformaldehyde in sea water (pH 7.4) at room temperature, followed by additional fixation in 2.5% glutaraldehyde/4% paraformaldehyde in sea water (pH 7.4) at room temperature for 1.5 h. After five 3 min washes in cold 0.1 M phosphate buffer (pH 7.4), samples were left overnight. The next day, they were incubated on ice for 60 min in 2% osmium tetroxide-1.5% potassium ferrocyanide-0.1 M PB-2 mM calcium chloride followed by five 3 min washes in distilled water at room temperature and then 20 min at room temperature in 0.22 μm Millipore filtered 1% thiocarbohydrazide in distilled water. After five 3 min washes in distilled water, they were incubated for 30 min in 2% osmium tetroxide in distilled water at room temperature. After five 3 min washes in distilled water, samples were exposed to aqueous 1% uranyl acetate at 4°C overnight. The next day, after five 3 min washes in distilled water, samples were exposed to 0.66% lead citrate-30 mM aspartic acid (pH 5.5) at 60°C for 30 min, washed five times for 3 min in distilled water at room temperature, and dehydrated through a graded series of aqueous ethanols to 95%. The coverslip with eggs was removed from the Petri dish and placed in an aluminum dish for washes with anhydrous 100% ethanol, two more washes in propylene oxide at room temperature, and a graded series of Durcupan infiltrations (according to the manufacturer’s directions; Sigma-Aldrich) for 2 h each up to 100% at room temperature, and incubation overnight with rotation. The next day, samples were incubated for 2 h in fresh Durcupan and finally cured in fresh resin at 60°C for 48–72 h.

### TEM

The coverslip was removed by thermal shock by liquid nitrogen dipping. The eggs of interest were located by comparison to the optical images acquired prior to embedding. The blocks were trimmed by a razor blade before diamond knife ultramicrotome (Leica UC7) sectioning 50–100 μm deep. One Pioloform-coated slot grid of four to eight 80 nm sections was collected followed by grids of 300 nm sections. A montage covering 25% of the egg was imaged (Tecnai 12 TEM, FEI) for the 80 nm sections for initial interpretation (data not shown). The 300 nm sections were coated with 25 nm gold fiducial markers (Aurion) followed by TEM tilt series tomography (Tecnai20 TEM, FEI; 200 keV) of spaces between yolk granules and any other features of interest. The single tilt series was taken ± 55° at 2° intervals at 2.1 nm/pixel. The series were aligned and back projection reconstructed (IMOD) prior to analysis in FIJI (35–37). A total of more than 20 tomograms were reconstructed with three to five representatives for each condition. The remaining resin blocks were prepared for SBF-SEM acquisition.

### SBF-SEM

The block was trimmed with a razor blade, removed, and mounted onto an aluminum pin using conductive epoxy glue (ITW Chemtronics, Enschede, The Netherlands) as previously described (38). The glue was then hardened at 60°C overnight and the block sputter coated with 2 nm platinum using a Q150R S sputter coater (Quorum Tech, East Sussex, UK).

**Fig. 4.**
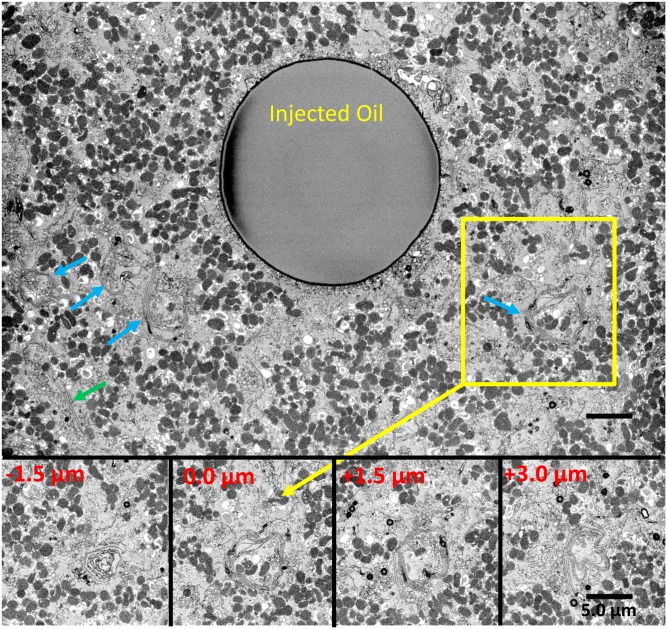
DGK-treated egg with forming coils. Egg contains many regions with varying degrees of sheet and coil formation (blue arrows indicate some coils and green arrow a flat stack of two membranes). One of the coil regions with multiple sheets was selected (yellow box) and images from the SBF-SEM stack are shown in the bottom insets at relative depths of −1.5, 0.0, +1.5, and +3.0 μm showing the complexity of thickness, shape, and sheet number and demonstrating a minimum length in the z direction of 4.5 μm for this part of the forming coil. The structures suggest that coils form progressively from stacks that feed into them (see supplemental Movie S3, which shows a complete z-series of the tilt series tomographic reconstruction of this region). Scale bar = 5.0 μm.

Correlative SBF-SEM data were collected using a 3View2XP (Gatan, Pleasanton, CA) attached to a Sigma VP scanning electron microscope (Zeiss, Cambridge) as previously described (38). The scanning electron microscope was operated at an accelerating voltage of 2 kV with high current mode active, using a 20 μm aperture and chamber pressure of ∼8 Pa. A per pixel dwell time of 2 μs was used with a slice thickness of 50 nm. Images were acquired at 8,192 × 8,192 pixels with 8.7 nm pixel resolution (horizontal frame width of 71 μm). The entire volume comprised 420–520 slices to give an ∼1,500 μm^3^ image volume as in Fig. 4.

## RESULTS

### Organelle morphology in eggs depleted of endogenous DAG

During sea urchin oogenesis, a period of active protein synthesis, the ER takes on several forms consistent with synthesis of yolk, cortical granule, lipid droplets, annulate lamellae, and organelle proteins (26). The mature oocyte, however, is characterized by very low rates of protein synthesis (39) and a random distribution of organelles except for cortical granules. Almost no rough ER remains from oogenesis and stacks of annulate lamellae occasionally appear bifurcated enclosing granular regions called “heavy bodies”.

We specifically labeled the ER by injection into the eggs of oil droplets containing the hydrophobic fluorescent dye, diIC_18_, which is rapidly depleted from the oil by ER membranes and equilibrates throughout the ER, thus demonstrating ER membrane continuity (11). The pattern of ER stained with diIC_18_ in an unperturbed control egg was stable for well over 5 h under our experimental conditions (**Fig. 1A**, B). It consists predominantly of a typical polygonal network of tubules connected at sites of three-way junctions, which surround organelles like yolk and mitochondria that appear in single confocal images as dark spots of varying diameters more or less uniformly distributed in the cytoplasm. The largest of these spots had a mean diameter of 1.52 ± 0.10 μm (n = 24), sufficient to enclose yolk or mitochondria (39). A few sheet-like structures believed to be stacks of annulate lamellae appeared in single optical sections as bright lines when seen on edge or occasionally broader structures (Fig. 1A–C). Electron microscopy confirmed the annulate lamellae (Fig. 1D–G, red arrows; see also supplemental Fig. S1 and supplemental Movie S1). Yolk and mitochondria distribution throughout the cytoplasm was fairly uniform. Yolk diameters from confocal images were 1.4–1.7 μm (n = 25). Diameters from EM (Fig. 1F, G, yellow arrows) were 1.0–1.7 μm (n = 28). Both maxima are consistent with known yolk sizes. Mitochondrial diameters were about half that size (Fig. 1F, G, green arrows). Overall, our electron micrographs of control unfertilized untreated oocytes are consistent with those of Verhey and Moyer (26).

However, in eggs depleted of DAG following microinjection of the enzyme DGK, which converts DAG to phosphatidic acid, bright fluorescent sheet regions in the confocal images accumulate and grow progressively (**Fig. 2A**, red arrows) as previously reported (20, 40). The ER remained interconnected as shown by diIC_18_ labeling after sheets begin to form (Fig. 2B; supplemental Movie S2), although the density of tubular structures decreased. The sheet regions contained bright lines characteristic of sheets on edge, circular, or elliptical structures and somewhat less intense regions, still brighter than tubules, that appear as facial views of sheets (**Fig. 3A**, B). Cylindrical structures (red arrows in Fig. 3B) extended more than 10 μm into the egg but could not be easily followed in the confocal z-stacks beyond this depth. Electron microscopy revealed the bright regions as mostly devoid of yolk but containing clustered mitochondria, perhaps a result of attachment of the ER to the latter (Fig. 3C–F). Membrane morphology of mitochondria, yolk, and the plasma membrane appeared to be largely unaffected compared with control eggs.

The induced ER sheets were of variable morphologies and were not annulate lamellae (Fig. 3). Sheets sometimes extended from annulate lamellae (Fig. 3C–E). These may be single or stacked, often bifurcate, and can form “onion skin-like” layered structures or cylinders of as many as eight or nine gently coiled layers with varying overall wall thickness dependent on the spacing between sheets (Fig. 3F). These structures in confocal images had various sizes and thicknesses and were generally elliptical in cross-section but cylindrical in 3D reconstructions. The apparent thickness of complete cylinder walls from seven eggs (26 cylinders) estimated from confocal microscopy varied from ∼0.5 to 1.0 μm. Their outside diameters varied from 3.4 to 13.7 μm.

Selected sections from a single region of this type from SBF-SEM extending over 4.5 μm in depth are shown in **Fig. 4**, showing that the number of contributing sheets was variable along the length of the coil (see also supplemental Movie S3). Some sheets extended into stacks beyond the coil, perhaps indicating a precursor relationship to forming coils.

**Fig. 5.**
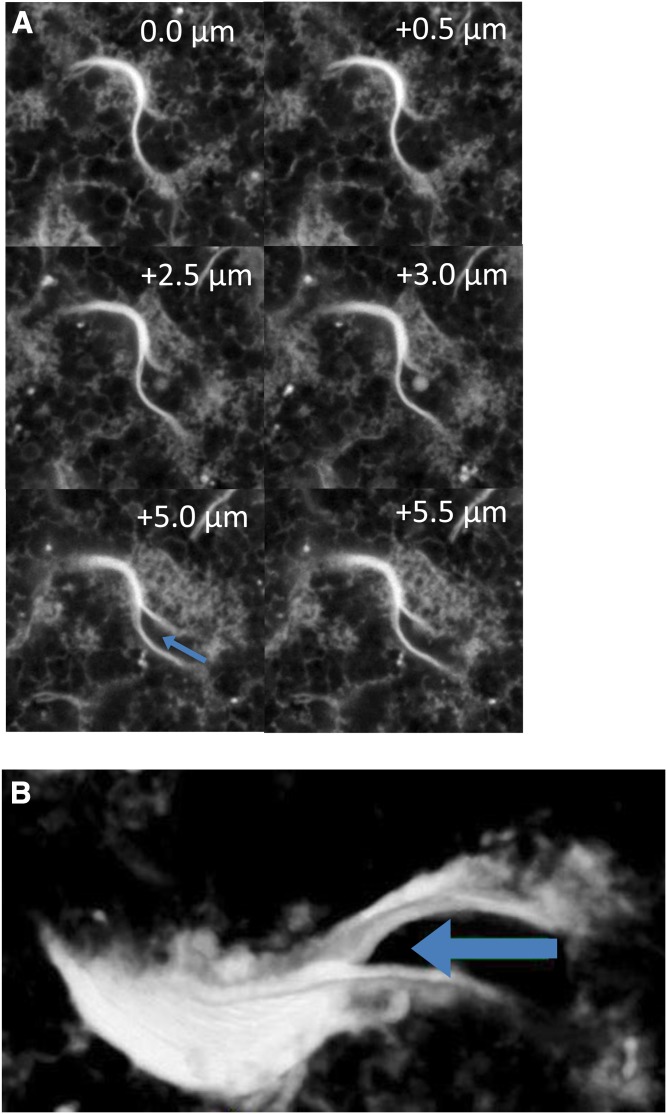
Confocal 3D reconstruction of bifurcated sheet region. A: Six confocal sections of a bifurcated sheet area taken from selected z-sections. B: 3D reconstruction of this region from 32 sections spaced at 0.5 μm intervals indicating a folding of the sheets into a partial coil. Arrow indicates region of bifurcation.

A region highlighting the potential relationship of stacks to an incipient coil is shown in **Fig. 5A**. Six selected confocal sections from a z-series showing a bifurcated curved stack extend 5.5 μm deep into the egg. This region was reconstructed from 32 such sections spaced at 0.5 μm intervals illustrating the complex topology of bifurcated sheets/stacks (Fig. 5B). We interpret this structure as a cup of stacked curved sheets splitting into two regions, which have not fully joined the cup that may itself be in the process of further folding into a coil (compare also the tan arrow in Fig. 3A and structures in the Fig. 4 insets).

Taken together, we interpret the confocal and electron microscope data to indicate that a fraction of the tubular network is converted to sheets and stacks when eggs are depleted of DAG. Conversion would relieve some constraints on the high degree of cross-sectional curvature characteristic of tubules.

### Kinetics of sheet area enlargement

In order to quantify the phospholipid dependence of the kinetics of sheet area formation for comparison between eggs in the same and different experiments, we subjected time lapse sequences of eggs to image analyses. Because sheets were mostly enclosed within a confocal section for some part of their length and several stacked sheets can contribute fluorescence within a given optical section and because yolk was excluded from sheet-rich regions thus blocking less of the emitted light, sheet regions were generally considerably brighter than tubule regions. Using image intensity as a criterion, sheet region areas and stacks could be readily distinguished from tubule regions (see Fig. 1C). Changes in the relative proportions of these structures could then be derived from measurements of their fractional cross-sectional areas relative to total ER. Although this method does not measure the actual fraction of membrane in the egg contributed by each morph, it permits a measure of the rate of interconversion of sheet regions for graphical comparison of eggs under differing experimental manipulations (40).

### Microinjections of DGK and PI-PLC act antagonistically on production of sheet areas

**Fig. 6.**
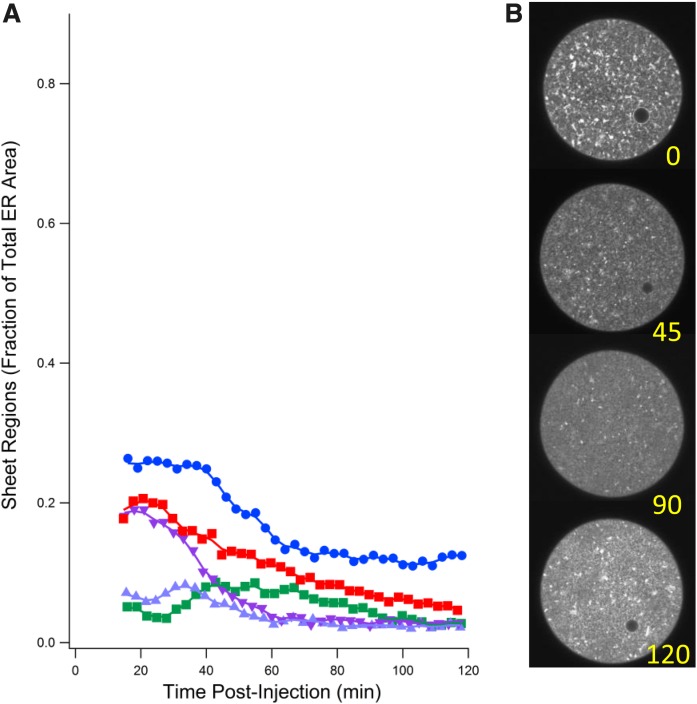
Effect of bacterial PI-PLC injection into unfertilized eggs. A: Decline in sheet regions of five eggs injected with PI-PLC. B: Successive images of an unfertilized egg injected with PI-PLC (200 μg/ml pipette concentration) showing slight decrease in sheet regions over time.

Because depleting endogenous 1,2-DAG with DGK (which converts DAG to phosphatidic acid) led to an increase in sheet areas, we tested to determine whether augmenting 1,2-DAG by microinjection of bacterial phospholipase C [which converts PtdIns(4,5)P_2_ to 1,2-DAG] would decrease sheet areas. We chose a bacterial PI-PLC that converts PtdIns to DAG but generates myo-inositol instead of inositol triphosphate in order not to alter internal Ca^2+^ concentration. When unfertilized eggs labeled with diIC_18_ were microinjected with this PI-PLC but no DGK, the enzyme rapidly drove the sheet fraction of ER cross-sectional areas close to minimum (**Fig. 6**).

**Fig. 7.**
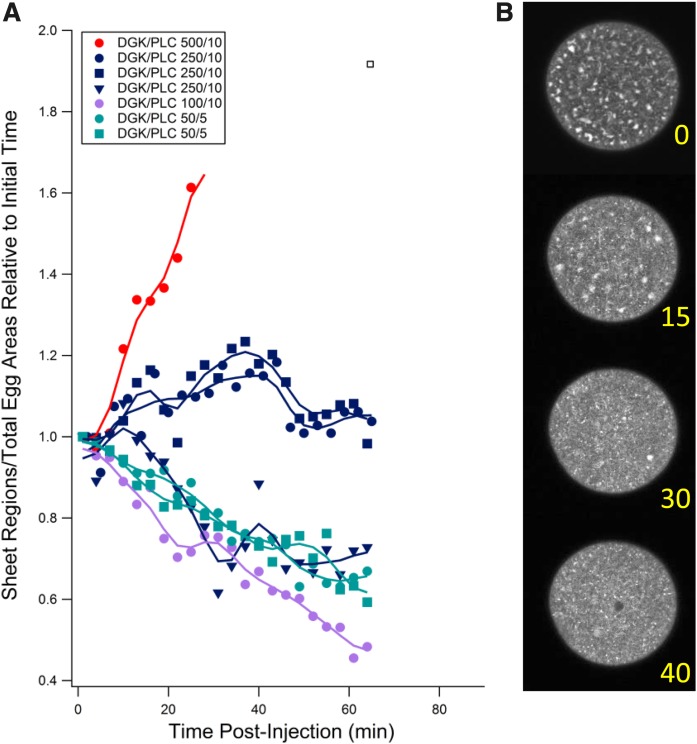
Effect of DGK/PI-PLC co-injection at various ratios on sheet region formation. A: Ratio of area of sheet regions to total egg area normalized to time of co-injection of DGK and bacterial phospholipase C (PI-PLC). Numbers indicate pipette concentrations in micrograms per milliliter of the two enzymes. Sheet regions accumulated at high DGK/PI-PLC ratios but were prevented or slightly reversed at lower ratios. B: Four images of one such egg injected at a 50/5 ratio showing decline in sheet areas over time (0–40 min).

We then tested to determine whether the PI-PLC and DGK activities could act antagonistically in the ER interconversion. Various ratios of DGK and PI-PLC were co-injected into unfertilized eggs and the fractions of sheet areas measured. **Figure 7** illustrates that at a DGK/PI-PLC ratio of 50:1, sheet areas rapidly increased. As the ratio was lowered to 25:1, sheet formation was effectively inhibited and, in one case, reversed toward tubule formation. At a 10:1 ratio, initial sheet areas decreased relative to the starting point. Thus, overproduction of DAG by PI-PLC leads to fewer sheet areas, and depletion of DAG by DGK leads to more sheet regions, the ratio depending on the relative total activities of the two enzymes.

### Effectiveness of phospholipids of varying degrees of negative spontaneous curvature on preventing sheet formation

To test the ability of unrelated lipids of different degrees of negative spontaneous curvature (1/R_o_p) to prevent sheet area formation, we preincubated eggs in a suspension of SUVs of varying composition to deliver excess lipids to the eggs prior to DGK injection and measured subsequent sheet area formation. SUVs were prepared by sonication of aqueous suspensions of phospholipids. A control experiment used SUVs containing only phosphatidylcholine (PtdCho), which has almost zero intrinsic negative spontaneous curvature [estimated at −0.0061 Å^−1^; see the table of spontaneous curvature in (41)]. PtdCho-containing SUVs had little or no effect on the progression of sheet formation (supplemental Fig. S2).

In contrast, SUV preincubation with SUVs containing either 1,3-DAG (a nonsignaling analog of 1,2-DAG, spontaneous curvature estimated at approximately −0.093 Å^−1^, not shown) or PtdEth (estimated at approximately −0.035 Å^−1^ to approximately −0.044 Å^−1^) was effective at preventing the formation of sheet areas by DGK (supplemental Fig. S2) (20, 40). Phosphatidic acid, the product of DGK phosphorylation of endogenous DAG, is estimated at −0.022 to −0.008 Å^−1^. Thus, the ability to prevent sheet area formation by phospholipids of negative spontaneous curvature correlates with their relative degree of negative spontaneous curvature.

### Reversal of sheet formation by exogenously added phospholipids with negative spontaneous curvature

To rule out the possibility that formation of sheet areas is an artifact related to irreversible destruction by DGK microinjection of the ability of the ER to organize properly, we tested the effectiveness of SUVs of intrinsic negative curvature to reverse the tubule to sheet interconversion. Approximately 20 min post-injection of DGK after sheet area formation was well underway, SUVs were added to the eggs and sheet formation was monitored.

**Fig. 8.**
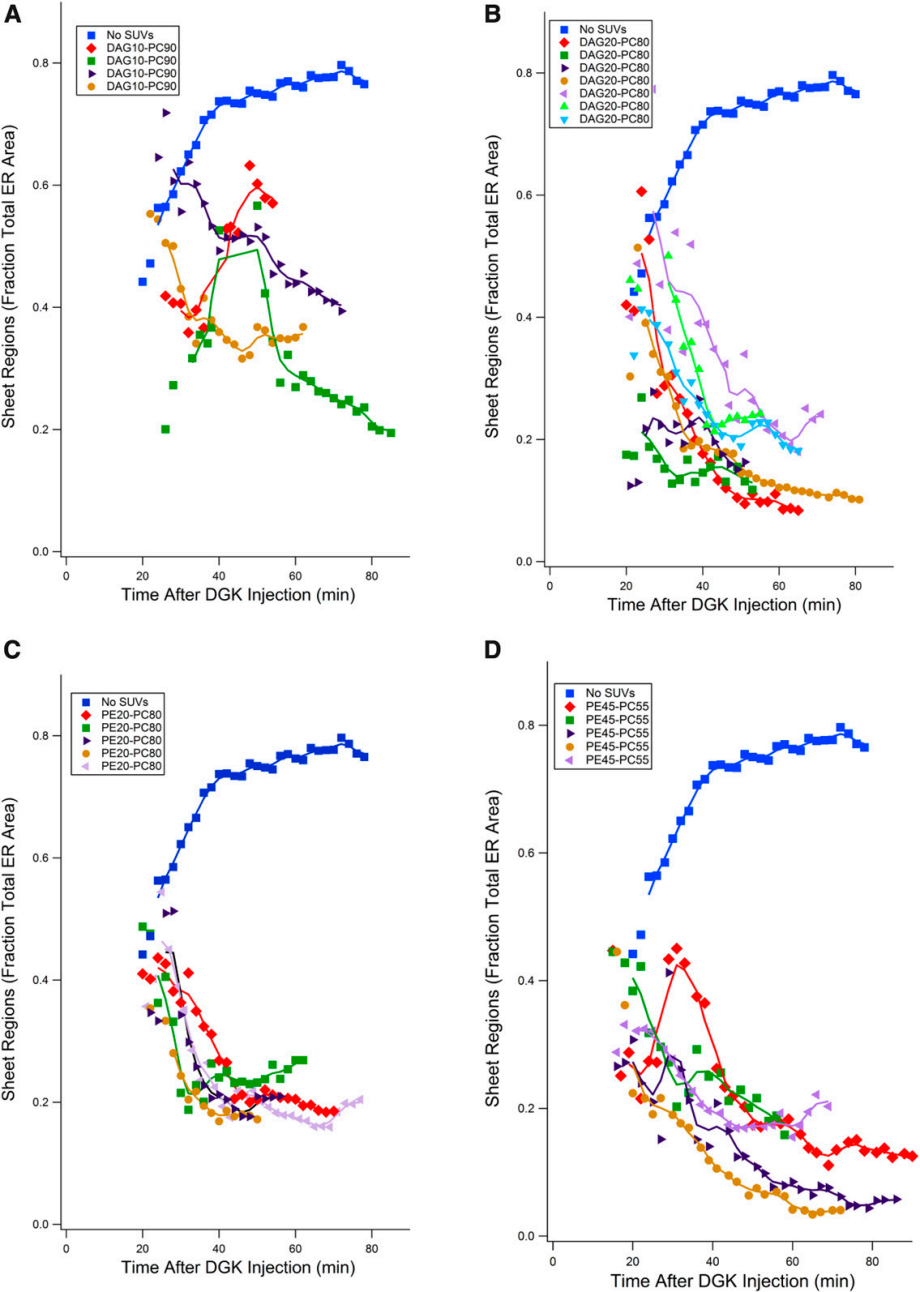
Ability of 1,3-DAG- or PE-containing SUVS to reverse sheet formation in DGK-injected eggs. A: 1,3-DAG (10 mol%)/PC (90 mol%), four eggs. B: 1,3-DAG (20 mol%)/PC (80 mol%), seven eggs. C: PE (20 mol%)/PC (80 mol%), five eggs. D: PE (45 mol%)/PC (55 mol%) PC. SUVs were added 15–20 min after DGK injection, five eggs. All four SUV preparations led to reversal of sheet formation. SUVs containing higher ratios of phospholipids with negative curvature were generally most effective.

SUVs containing 10 or 20 mol% 1,3-DAG were capable of reversing sheet formation and returning sheets to near initial values (**Fig. 8A**, B). Ten percent 1,3-DAG was not as effective as 20% in the extent of reversal by 80 min and sometimes showed a lag period. Twenty percent 1,3-DAG SUVs drove down sheet areas more quickly and to a greater extent. Because 1,3 DAG does not bind to signaling molecules like protein kinase C (42), this suggests that the effect was not through signaling pathways but a direct structural outcome.

We also tested PtdEth at 20 and 45 mol%, both of which were effective at reversing sheet formation (Fig. 8C, D). SUVs of 20% PtdEth drove sheet areas down somewhat less effectively than 20% DAG, but 45% acted similarly to 20% DAG. PtdEth does not share the immediate metabolic pathways of DAG, suggesting that the effect of depleting 1,2-DAG with DGK was not due to disruption of PtdIns pathway interconversions.

Therefore, the degree of sheet area formation depends reversibly on the amount of phospholipids of negative spontaneous curvature delivered to the cell.

### Cytoskeletal disruptors do not lead to sheet area formation

Microtubules are not necessary for ER tubule reticular formation in vitro (43), and maintenance of peripheral tubules in cultured mammalian cells is not necessarily immediately dependent on microtubule integrity (16) but microtubules are needed to extend ER tubules. Actin microfilament depolymerization has been reported to yield more endoplasmic reticular structures, and microtubule depolymerization more sheets, at the cell periphery of cultured human cells, suggesting that microfilaments might act antagonistically with microtubules (44, 45).

Unfertilized sea urchin eggs contain very few organized microtubules compared with fertilized eggs (46), and microfilament structure is largely located cortically until cytokinesis (47). Nonetheless, to test to determine whether lipid alteration was indirectly leading to sheet area formation by disruption of cytoskeletal elements, we exposed unfertilized eggs to either colcemid or cytochalasin D at concentrations known to disrupt microtubules or microfilaments, respectively, in sea urchin eggs (30, 32).

A 10 min treatment with 5 μM of colcemid blocks microtubules for 2 h in sea urchin eggs. We treated for 15 min or continuously for 2 h with no increase of sheet regions (supplemental Fig. S3A). Similarly, a 15 min pulse of cytochalasin D at 4 μg/ml, conditions that disrupt microfilaments, did not lead to formation of sheet regions. Cells could be exposed continuously to 4 μg/ml cytochalasin D for 45 min without formation of sheet regions, although for longer times, eggs show signs of general deterioration and burst, likely due to severe destruction of cell cortex organization. Therefore, sheet region formation does not appear to result from a disruption of microtubules or actin microfilaments in sea urchin oocytes.

### Protein synthesis inhibition has no effect on sheet area formation

To test to determine whether sheets formed because of an indirect effect of DGK due to a lack of synthesis of proteins needed to maintain tubules in the egg, eggs were incubated in 100 μM of emetine for 30 min, after which eggs were injected with DGK. In the absence of new protein synthesis, eggs nonetheless formed sheet areas (supplemental Fig. S3B). Control exposures to emetine without DGK injection did not result in production of sheet areas. Therefore, lack of sufficient synthesis of new ER tubule-shaping proteins as an explanation of sheet area formation in our experiments is not likely.

## DISCUSSION

The relationship between ER structure and function is now well-established. Several diseases have been correlated with alterations of structure and structural proteins that shape the ER by bending or spacing its membranes. The first order of organization of cellular membranes is the phospholipid bilayer with possible contributions of non-bilayer regions. Together these provide the context for the increasingly well-characterized structural proteins to act. However, as yet, relatively little attention has been paid to how the lipid composition of the membranes might affect the ability of these proteins to shape structure and control function.

Bending of cellular membranes defines the shape of organelles (as well as being essential to fusion and fission of membranous compartments that contribute to macromolecular traffic and organelle biogenesis and remodeling). ER sheets are much less curved than tubule cross-sections except at their tips (48). Small vesicles, edges of ER sheets and Golgi stacks, and the cross-sections of ER tubules all exhibit regions of much higher curvature in contrast to plasma membrane, nuclear membranes, and most of ER sheets.

Extreme bending of cellular phospholipid bilayers is energetically unfavorable and has been shown to depend on association with intrinsic or extrinsic proteins associated with the ER. Bending should also depend on phospholipid composition, which could be asymmetric across the bilayer or favor nonlamellar phases, such as inverted hexagonal (H_II_), each of which can make bending of pure phospholipid membranes energetically more favorable. Membrane curvature (49) can depend on asymmetry across the bilayer of both proteins and lipids of differing spontaneous curvature. Spontaneous curvature is influenced primarily by the head group but also by the degree of saturation and acyl chain length (50, 51).

We therefore acutely perturbed the lipid composition of ER membranes in a relatively inert cell to see how ER morphology would be affected. We chose sea urchin oocytes because of their low rates of protein synthesis, initial predominantly tubular ER structure, size, and clarity. Because the overall morphology of the ER in sea urchin oocytes is stable for long periods, but can be rapidly remodeled by the cell within minutes of fertilization, the cells provide a potentially dynamic system for structural rearrangements and response to perturbation.

Depletion of endogenous DAG by microinjection of DGK or by a rapalog dimerization device in the echinoderm or mammalian cells led within minutes to depletion of tubules and increases in large sheet-containing regions (20, 52). Here, we characterize the sheet regions in detail by confocal microscopy, transmission microscopy, electron tomography, and SBF-SEM on living and fixed eggs. We show that the resultant sheets were often stacked in large arrays or gently coiled structures. The ER membrane was particularly sensitive to DAG depletion, because yolk, mitochondrial, cortical granule, and plasma membranes appeared unaltered. Sometimes the nuclear envelope shape was altered, but this structure is continuous with and can be considered part of the ER (see supplemental Movie S2). ER sheets stacked into more or less flat layers or large gently coiled multilayered structures. Sheets were sometimes seen to be continuous with annulate lamellae.

Formation of sheet regions could be prevented by treatment of eggs with lipids of negative spontaneous curvature, PtdEth, or 1,3-DAG, a nonsignaling isomer of 1,2-DAG, prior to DGK injection (42), but not by PtdCho, a lipid displaying no negative spontaneous curvature. We also show that these regions could be reversed once they began to form by the same lipids, indicating that sheet formation was not due to the inability of the cells to form and maintain ER tubules. Furthermore, we demonstrate that DGK and bacterial PI-PLC, which respectively deplete and form 1,2-DAG in vivo, acted antagonistically in formation of these regions. (The bacterial PI-PLC does not produce IP_3_, a potent Ca^2+^ trigger in eukaryotic cells, thus eliminating a side effect of the enzyme.) Inhibition of protein synthesis did not significantly alter sheet formation, indicating that changes in morphology do not require synthesis of additional shape-forming proteins. Inhibitor experiments showed that sheet formation was not apparently a consequence of depolymerization of microtubules or microfilaments. Thus the levels of endogenous DAG and other lipids contribute to the maintenance of ER structure.

Our experiments do not distinguish between various molecular models of the action of the phospholipids. The theoretical basis for contributions of lipids of negative spontaneous curvature to overall membrane curvature was noted in models of membrane fusion intermediates, which exhibit high degrees of curvature in their fusion stalk regions. Evidence suggests that these phospholipids promote fusion of natural and synthetic protein-free membranes, whereas lipids of positive spontaneous curvature inhibit fusion (53–57). Asymmetric disposition of lipids (such as DAG and PtdEth with negative spontaneous curvature) can favor differential monolayer curvature leading to bending of the stalks based on molecular packing parameters or a bending moment of the monolayer.

Additionally, certain lipids (like DAG and PtdEth) favor nonlamellar packing such as HII phases, which may contribute to transient microdomains, for example, at membrane fusion stalks (53). In vitro fusion reactions of liposomes of varying lipid composition mediated by the GTPase, Sey1p (a yeast atlastin ortholog), was not strongly dependent on specific lipids for Sey1P docking, but lipid mixing (fusion) was strongly dependent on PtdEth or the cholesterol analog ergosterol, which both exhibit negative spontaneous curvature (23). Also, fusion of cortical granules with the plasma membrane in sea urchin eggs was strongly dependent on cholesterol, which could be substituted by other lipids of high negative spontaneous curvature, such as DAG or PtdEth (41).

Phospholipids could also exert their effects either by interacting directly with membrane-shaping proteins or through more long range structural effects (17, 58). Specific lipids can bind to proteins to confer conformational changes or serve as anchors. Moreover, the physical properties of the lipid bilayer (curvature stress field) can alter the behavior of proteins (17, 53). Long-range properties of the bilayer, such as elasticity, have been incorporated into a curvature force field (FSM) model for regulation of rhodopsin function (59). FSM considers minimization of hydrophobic solvation energy by either bilayer or protein deformation.

That in our experiments DAG and PtdEth had similar effects suggests that their shared physical properties, such as lipid shape or the ability to contribute to nonlamellar structures, rather than specific chemical interactions with proteins or disruption of their metabolic pathways, are responsible for the morphological changes we detected. Whether one or more of the discussed mechanisms is responsible for the sensitivity of ER shape to lipid composition, we believe it important to interpret the effects of shaping proteins in the context of the immediate and variable lipid environment of those proteins.

With the further development of techniques to assay lipid levels at specific locations within individual cells, the experimental system used here should provide a model for quantification and localization of lipid changes in vivo under altered metabolic or experimental conditions. It is also worth noting that the extensive and rapid changes in ER structure, which depend on large scale disruption of phospholipid content that we report here, suggest that attention should be paid to perhaps less extensive lipid deficiencies as causes and therapeutic targets in a number of diseases associated with ER stress. Future studies with emergent techniques of greater precision in determining the spatial distribution and localized levels of individual lipids will be needed to further clarify their putative physiological roles at sites of bending that lead to shape changes.

## SUMMARY

Changes in the complex morphology of the ER are found in a number of diseases. This morphology is dependent not only on shaping proteins but also on adequate levels of specific lipids.

## Supplementary Material

Supplemental Data
