# Supplementary material for: Lipid species affect morphology of endoplasmic reticulum: a sea urchin oocyte model of reversible manipulation
Source: J Lipid Res. 2019 Sep 23;60(11):1880–91. doi: 10.1194/jlr.RA119000210 (PMC6824487; doi:10.1194/jlr.RA119000210)
Supplement: Supplemental Data [file supp_60_11_1880__index.html]

Lipid species affect morphology of endoplasmic reticulum: a sea urchin oocyte model of reversible manipulation — Lipid alterations and reversible changes in ER morphology — Lipid species affect morphology of endoplasmic reticulum: a sea urchin oocyte model of reversible manipulation — Supplemental Data 

# Lipid species affect morphology of endoplasmic reticulum: a sea urchin oocyte model of reversible manipulation

## Supplemental Data

- Supplemental Figure 1. Examples of ER structures . - A) Tomographic reconstruction from an uninjected egg (see Figure 1G). B,C ) Tomographic reconstructions from an egg after DGK injection (see Figure 3D,E). i ) Annulate lamellae (yellow arrow), note the regular spacing of the &#x201C;pores&#x201D; connected by the two membranes characteristic of AL. The &#x201C;pore&#x201D; diameters were 77 27nm (mean &#x00B1;S.D .). ii) Sheets (blue arrows), two membrane ER stacks with a continuous separation space approximately between each differing from annulate lamellae in lack of &#x201C;pores&#x201D;. iii iv) Annulate lamellae or possibly fenestrated sheets sometimes connected at their ends to single sheets. v) is similar to iii iv but from an orthogonal view. vi) tubular ER with greater distance between membranes compared to ii which do not appear vesicular from successive focal planes but may represent swollen less and therefore less curved tubules due to DGK treatment.
- Supplemental Figure S2. Ability of Exogenous PE but not PC Containing SUV Pre incubation to Prevent Sheet Region Formation by DGK. - Eggs pre-incubated with SUVs containing 20 mole % PE/80 mole % PC (DGK PE 20) blocked formation of sheets by microinjected DGK. 100 mole % PC SUVs (DGK PC 100) did not prevent sheet formation by DGK. &#x00B1;S.D, n=5 for No DGK PC100, No DGK No SUV and DGK PE20.
- Supplemental Figure S3. Effects of Microtubule and Actin Microfilament Disruption and Protein Synthesis Inhibition on Sheet Region Formation. - A) Continuous or pulsed exposure to cytochalasinD (4 &#x03BC;g/ml) n=5 or colcemid(5 &#x03BC;M). n=7; &#x00B1;S.D. Cytochalasin pulse was 15 min with 2 min washout prior to data collection. B) Inability of protein synthesis inhibitor emetine (100 &#x03BC;g/ml) to block sheet formation by DGK (7 &#x03BC;g/ml). Emetine was added 20 min after DGK or buffer injection. Data indicate that disruption of cytoskeletons does not result in sheet area formation and new protein synthesis is not required for DGK to form sheet regions.
- Supplemental Movie S1. 3-D Reconstruction of Control Egg - Complete Z-series of the tilt series tomographic reconstruction of Figure 1G.
- DGK-Injected Egg During Sheet Forming - Supplemental Movie S2. Egg injected with DGK and imaged 20 min later showing ER continuity.
- Supplemental Movie S3. Video of Data Stack for Figure 4 of a DAG-Depleted Egg - DAG Depleted Egg
