# Supplementary material for: Lipid species affect morphology of endoplasmic reticulum: a sea urchin oocyte model of reversible manipulation
Source: J Lipid Res. 2019 Sep 23;60(11):1880–91. doi: 10.1194/jlr.RA119000210 (PMC6824487; doi:10.1194/jlr.RA119000210)
Supplement: Supplemental Data [file supp_RA119000210_153626_1_supp_393420_pxssgs.pdf]

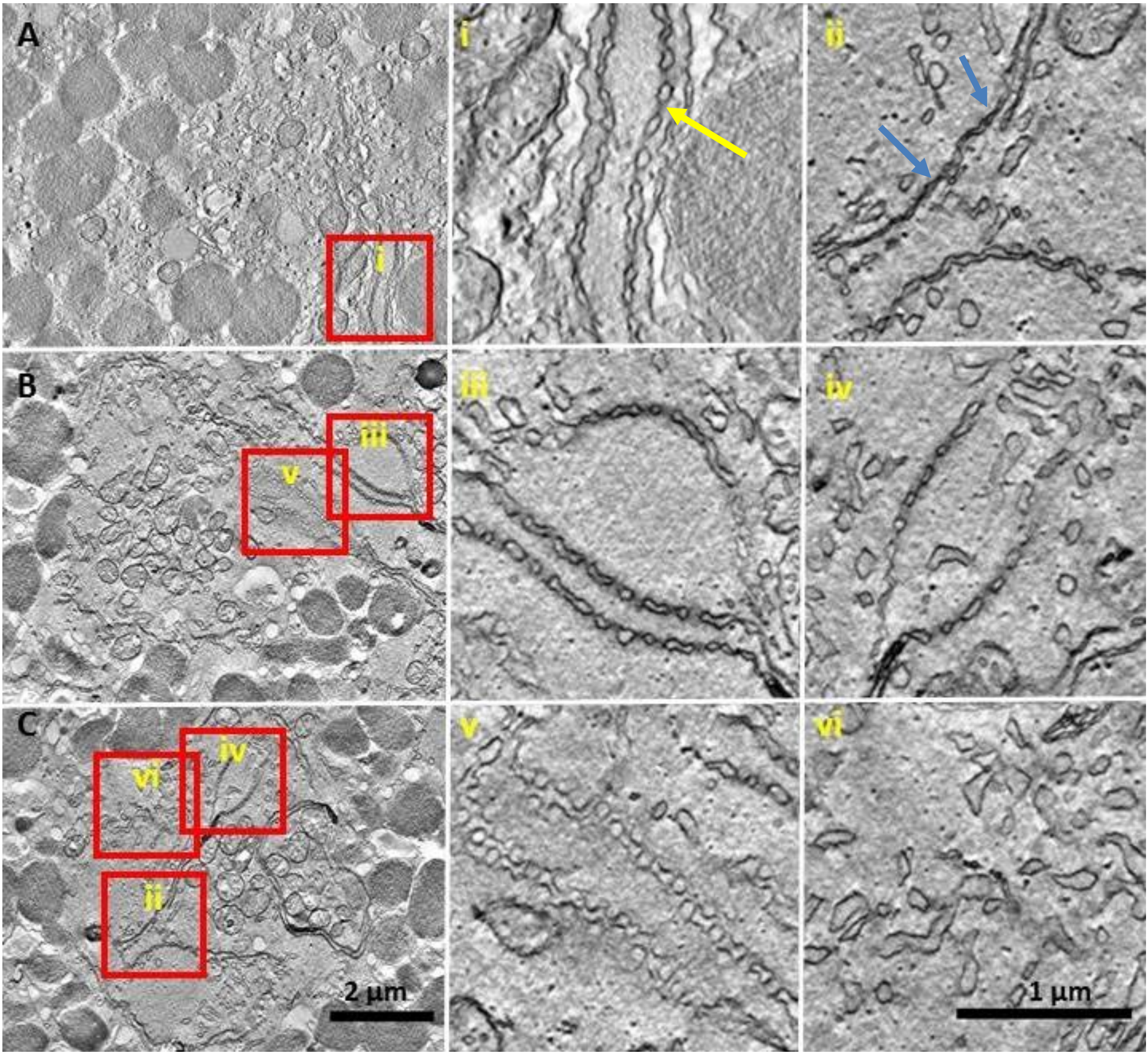

Figure S1. Examples of ER structures. A) Tomographic reconstruction from an uninjected egg (see Figure 1G). B,C) Tomographic reconstructions from an egg after DGK injection (see Figure 3D,E). i) Annulate lamellae (yellow arrow), note the regular spacing of the "pores" connected by the two membranes characteristic of AL. The "pore" diameters were 77±27nm (mean±S.D.). ii) Sheets (blue arrows), two membrane ER stacks with a continuous separation space approximately between each differing from annulate lamellae in lack of "pores". iii-iv) Annulate lamellae or possibly fenestrated sheets sometimes connected at their ends to single sheets. v) is similar to iii-iv but from an orthogonal view. vi) tubular ER with greater distance between membranes compared to ii which do not appear vesicular from successive focal planes but may represent swollen less and therefore less curved tubules due to DGK treatment.
