# Supplementary material for: Lipid species affect morphology of endoplasmic reticulum: a sea urchin oocyte model of reversible manipulation
Source: J Lipid Res. 2019 Sep 23;60(11):1880–91. doi: 10.1194/jlr.RA119000210 (PMC6824487; doi:10.1194/jlr.RA119000210)
Supplement: Supplemental Data [file supp_RA119000210_153626_1_supp_393421_pxnngn.pdf]

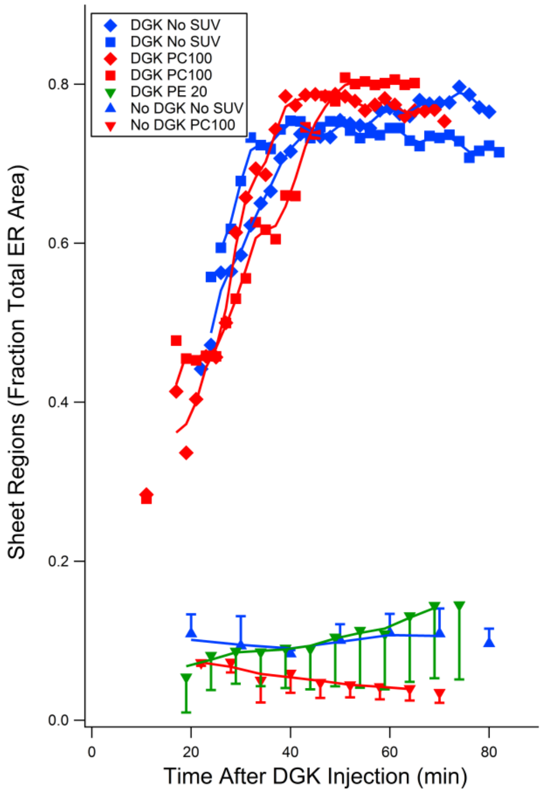

Figure S2. Ability of Exogenous PE- but not PC-Containing SUV Pre-incubation to Prevent Sheet Region Formation by DGK. Eggs pre-incubated with SUVs containing 20 mole % PE/80 mole % PC (DGK PE 20) blocked formation of sheets by microinjected DGK. 100 mole % PC SUVs (DGK PC 100) did not prevent sheet formation by DGK. ±S.D, n=5 for No DGK PC100, No DGK No SUV and DGK PE20.
