# Supplementary material for: Lipid species affect morphology of endoplasmic reticulum: a sea urchin oocyte model of reversible manipulation
Source: J Lipid Res. 2019 Sep 23;60(11):1880–91. doi: 10.1194/jlr.RA119000210 (PMC6824487; doi:10.1194/jlr.RA119000210)
Supplement: Supplemental Data [file supp_RA119000210_153626_1_supp_393422_px22g2.pdf]

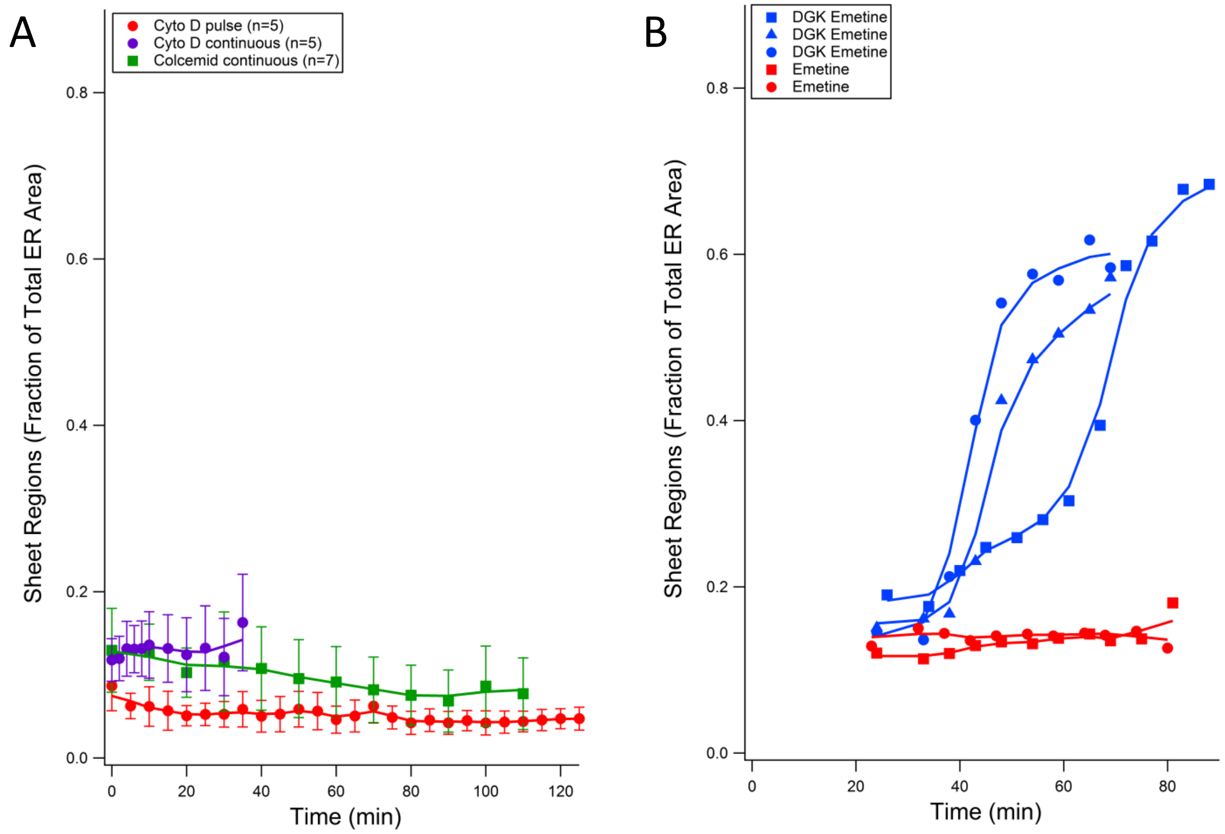

Figure S3. Effects of Microtubule and Actin Microfilament Disruption and Protein Synthesis Inhibition on Sheet Region Formation. A) Continuous or pulsed exposure to cytochalasin D (4 µg/ml) n=5 or colcemid (5 µM). n=7; ±S.D. Cytochalasin pulse was 15 min with 2 min washout prior to data collection. B) Inability of protein synthesis inhibitor emetine (100 µg/ml) to block sheet formation by DGK (7 µg/ml). Emetine was added 20 min after DGK or buffer injection. Data indicate that disruption of cytoskeletons does not result in sheet area formation and new protein synthesis is not required for DGK to form sheet regions.
